# Early identification of brain injury in infants with hypoxic ischemic encephalopathy at high risk for severe impairments: accuracy of MRI performed in the first days of life

**DOI:** 10.1186/1471-2431-14-177

**Published:** 2014-07-08

**Authors:** Thais Agut, Marisol León, Mónica Rebollo, Jordi Muchart, Gemma Arca, Alfredo Garcia-Alix

**Affiliations:** 1 Deparment of Neonatology, Agrupació Sanitaria Hospital Sant Joan de Déu-Hospital Clinic-Maternitat, University of Barcelona, Barcelona, Spain; 2 Department of Neuroradiology, Hospital Sant Joan de Déu, University of Barcelona, Barcelona, Spain

**Keywords:** Hypoxic-ischemic encephalopathy, MRI, Newborn, Brain injury, Hypothermia, Sequential MR studies

## Abstract

**Background:**

Despite therapeutic hypothermia 30-70% of newborns with moderate or severe hypoxic ischemic encephalopathy will die or survive with significant long-term impairments. Magnetic resonance imaging (MRI) in the first days of life is being used for early identification of these infants and end of life decisions are relying more and more on it. The purpose of this study was to evaluate how MRI performed around day 4 of life correlates with the ones obtained in the second week of life in infants with hypoxic-ischemic encephalopathy (HIE) treated with hypothermia.

**Methods:**

Prospective observational cohort study between April 2009 and July 2011. Consecutive newborns with HIE evaluated for therapeutic hypothermia were included. Two sequential MR studies were performed: an •early’ study around the 4th day of life and a •late’ study during the second week of life. MRI were assessed and scored by two neuroradiologists who were blinded to the clinical condition of the infants.

**Results:**

Forty-eight MRI scans were obtained in the 40 newborns. Fifteen infants underwent two sequential MR scans. The localization, extension and severity of hypoxic-ischemic injury in early and late scans were highly correlated. Hypoxic-ischemic injury scores from conventional sequences (T1/T2) in the early MRI correlated with the scores of the late MRI (Spearman ρ = 0.940; p < .001) as did the scores between diffusion-weighted images in early scans and conventional images in late MR studies (Spearman ρ = 0.866; p < .001). There were no significant differences in MR images between the two sequential scans.

**Conclusions:**

MRI in the first days of life may be a useful prognostic tool for clinicians and can help parents and neonatologist in medical decisions, as it highly depicts hypoxic-ischemic brain injury seen in scans performed around the second week of life.

## Background

Hypoxic-ischemic encephalopathy (HIE) secondary to perinatal asphyxia remains a major cause of neonatal mortality and morbidity worldwide. Randomised control trials of therapeutic hypothermia (TH) for HIE have demonstrated a reduction in death or severe disability at 18 months of age. However, death and disability continue to occur in 30% to 70% of infants with moderate-to-severe encephalopathy despite treatment with cooling [1-8]. The localization, distribution and severity of hypoxic-ischemic lesions detected by MRI can be graded and related to outcome. MRI performed in the second week of life predicts outcome in infants with HIE [9-12]. However, one issue that remains unanswered is if early MRI, performed in the first days of life, in newborns treated with therapeutic hypothermia reflects brain hypoxic-ischemic damage in all its extension [13]. This question is crucial since there is a need for an early and accurate identification of infants who will have very severe impairment if they survive. As reported in the recent cooling trials, two-thirds of deaths in HIE infants followed withdrawal of life sustaining treatment [4,5]. If decisions are delayed, there is a possibility that the infant will survive with very severe long-term disabilities. As end of life decisions are relying more and more on the results of MRI performed in the first days of life [14], it is essential to determine whether early MRI findings reflect brain damage seen in later MRI in newborns with HIE treated with hypothermia. As far as we know there is only one study comparing images in early MR studies with the ones in scans performed in the second week of life [13].

The aim of this study was to evaluate how MR images performed around day 4 of life (“early MR”) correlate with the ones obtained in the second week of life (“late MR”) in infants with hypoxic-ischemic encephalopathy treated with hypothermia. Two sequential MR studies were performed to test this hypothesis.

## Methods

This is a substudy of a prospective observational cohort study that was conducted at Agrupació Sanitaria Sant Joan de Déu-Clinic-Maternitat Hospital in Barcelona, Spain between April 2009 and July 2011. Consecutive asphyxiated newborns with a gestational age ≥35 weeks admitted to the level III neonatal intensive care unit to be evaluated for therapeutic hypothermia were included. Our eligibility criteria were the following: (1) Evidence of fetal distress, with at least one of the following: a) Apgar score at 5 min ≤ 5; b) continued need for ventilation initiated at birth for at least 10 min; c) pH ≤ 7.00 in arterial cord blood or other blood sample in the first hour of life and, (2) evidence of moderate or severe neonatal encephalopathy in the first 6 hours of life. Encephalopathy was classified in mild, moderate or severe according to a previously reported scale. This Classification system is a modification of the grading system described by Sarnat that focuses in the level of alertness. It sub classifies moderate HIE in A or B if seizures are present or absent respectively and, severe HIE in A or B if brain function is preserved or abnormal respectively [15].

Newborns who met criteria for moderate or severe HIE received whole-body cooling within 6 hours of life. Whole-body cooling was achieved with a blanket-cooling device (Techotherm TS med 200 N Olympic^®^) regulated by the infant’s core temperature measured with a rectal probe. Neonates were maintained at 33.5°C for 72 hours and were then slowly rewarmed (≤ 0.5°C per hour) and monitored with amplitude-integrated electroencephalography (aEEG) during the entire process.

Clinical data were prospectively collected including information regarding prenatal, perinatal and postnatal variables. Informed parental consent was obtained and the study was approved by the Research and Ethics Committee at Sant Joan de Deu’s Hospital.

Of the 62 neonates born or referred to our Unit for evaluation of encephalopathy and possible therapeutic hypothermia, five were excluded because they had other diagnoses rather than HIE. Of the 57 newborns with perinatal HIE two were excluded for late referral (after 6 hours of age), three because they were premature newborns less than 35 weeks gestational age and one whose parents refused to participate in the study, resulting in 51 newborns with perinatal HIE (11 mild, 15 moderate and 25 severe).

### MR imaging

Two sequential MR studies were included in the imaging protocol: an•early’ study performed around the 4th day of life and a •late’ study during the second week of life.

Standard MRI was performed using 1,5 Tesla units (General Electrics). A specific neonatal head coil was used and the following imaging protocol performed: axial 3D FSPGR IR (TR 12/TE 5/IR 450/1.4 mm), axial T2 FSE (TR 3500/TE 91/5.5 mm), T1 sagittal FLAIR (TR 2500/TE 24/TI 750/5 mm), diffusion weighted images (DWI) axial (b = 0/1000 /TR 8000/TE 91/5 mm/3 directions). A neonatologist was present throughout the procedure and heart rate and transcutaneous oxygen saturation were monitored using a pulse oximeter. Passive hypothermia was maintained during the early brain MR scans when performed before rewarming without any adverse events.

Newborns were grouped into 4 patterns of injury on the basis of the predominant site of injury on MRI: normal, basal ganglia/thalamus injury, watershed pattern, and global injury [16]. MR studies were assessed independently by two neuroradiologists (JM and MR), who were unaware of the number of the study and blinded to the clinical condition of the infants. Conventional (T1 and T2) and diffusion images were scored separately according to the scheme described by Rutherford [12,17]. Discrepancies in the scoring of the images were discussed and resolved by consensus. Images were examined for normal anatomic development and for the presence of abnormal signal intensities within the basal ganglia and thalamus (BGT), posterior limb of the internal capsule (PLIC), white matter (WM) and cortex (sites documented included specifically the central sulcus, interhemispheric fissure, and the insula).

### Statistical analysis

The data were analyzed using SPSS version 20 (SPSS, IL, USA). Clinical variables were compared using the appropriate test (Χ^2^, Fisher exact test for categorical variables, and Mann–Whitney or Kruskal–Wallis test for continuous data). Relationship between MR scores in the first and second scans was assessed using Spearman’s rank correlation coefficient (ρ). Differences with p-level < 0.05 were considered statistically significant.

## Results

Forty of the 51 infants with HIE had a moderate or severe encephalopathy and were treated with hypothermia: encephalopathy was classified as moderate in 15 (37.5%) and severe in 25 (62.5%). General perinatal data are presented in Table 1. There were no significant differences in the perinatal data between the two groups except for the need of a more advance resuscitation in infants with severe HIE. Sixteen patients (40%), one with moderate and 15 with severe HIE died during the neonatal period.

**Table 1:** Perinatal characteristics of HIE newborns treated with hypothermia

	**Moderate HIE (n = 15)**	**Severe HIE (n = 25)**	**p**
GA (wks) mean ± SD	39.3 ± 1,5	38.4 ± 2.2	0.179
Birth weight (g) media ± DE	3108 ± 594	2981 ± 583	0.512
Male infants N (%)	10 (66.7)	15 (60)	0.746
Inborn N (%)	4 (26.7)	4 (16)	0.444
Abnormal FHR N (%)	7 (46.7)	14 (56)	0.750
Sentinel event N (%)	7 (46.7)	8 (33.3)	0.505
Emergency CS N (%)	13 (86.7)	25 (100)	0.135
Vertex presentation N (%)	15 (100)	21 (87.5)	0.271
pH^1^ mean ± SD	6.90 ± 0.13	6.90 ± 0.21	0.940
Base deficit mean ± SD	20.22 ± 5.34	20.41 ± 5.97	0.922
Lactate mean ± SD	13.82 ± 5.53	16.44 ± 5.45	0.277
1-min Apgar score median (range)	1 (0–4)	2 (0–6)	0.638
5- min Apgar score median (range)	4 (2–9)	4 (0–8)	0.099
10- min Apgar score median (range)	6 (3–9)	7 (0–10)	0.624
Advance resuscitation^2^ N (%)	6 (40)	20 (80)	0.017
Neonatal death N (%)	1 (6.7)	15 (60)	0.001

### MR imaging

Forty-eight MRI scans were obtained in the 40 newborns infants with moderate-severe HIE: 28 early and 20 late MR studies. Most of the infants (33/40) had at least one MR scan. Six patients died before an MR scan could be performed and in one patient the MR study was not available for assessment. The neonatal characteristics were similar in the group with and without MR study except that mortality, as expected, was higher in the latter group (p = 0.008). Of the 33 infants who underwent MRI, 23 had brain injury detected on MRI (69.7%). According to the severity of the encephalopathy, 5 out of 14 in the moderate group (35.7%) and 18 out of 19 in the severe (94.7%) showed abnormalities in their scans. The most frequent pattern of hypoxic-ischemic injury in newborns with moderate HIE was the (BGT) pattern whereas in newborns with severe HI was the global. Watershed injury was only present in two infants, both with severe HIE. Another infant with severe encephalopathy showed multiple punctate lesions in periventricular white matter (Table 2). We have not found any relationship between the patterns of injury and perinatal variables such as the presence of sentinel events (data not shown).

**Table 2:** MR findings in HIE newborns treated with hypothermia

		**Moderate HIE (n = 14)**	**Severe HIE (N=19)**	**Total (N = 33)**
Normal MRI		9	1	10
Abnormal MRI		5	18	23
Pattern	Central	3	4	7
	Global	2	11	13
	Watershed	0	2	2
	Punctata	0	1	1

### “Early versus late MR imaging”

Fifteen infants finally underwent two sequential MR scans, mainly because six infants died before an early scan was performed and in 5 it was not possible to obtain due to clinical instability. There were no differences between these infants and the rest of the cohort in terms of maternal, antenatal, or perinatal factors. Early scans were performed between the 2nd and 5th day of life and late scans between the 8th and 15th day of life. The average age (mean ± SD) at which early scans were obtained was 98.7 ± 26.7 hours in the moderate HIE infants and 90.4 ± 31.1 hours in the severe ones. For the late scans the average age was 303.2 ± 59.7 and 274.6 ± 62.2, respectively. There was no difference in the age at which the MR scan was obtained between the two groups. The pattern of injury and scores for each patient who underwent early and late MR studies are detailed in Table 3.

**Table 3:** Hypoxic-ischemic injury in newborns with two sequential scans

**Case number**	**Graduation of HIE**	**Pattern injury**	**Age MR1 (hrs.)**	**Score MR1**	**Score DWI**	**Age MR2 (hrs.)**	**Score MR2**
1	Moderate (B)	No injury	114	0	0	331	0
2	Moderate (B)	No injury	74 (HT)	0	0	362	0
3	Moderate (B)	No injury	91	0	0	402	0
4	Moderate (B)	No injury	115	0	0	238	0
5	Moderate (B)	No injury	68 (HT)	0	0	335	0
6	Moderate (A)	Central	95	4	4	189	6
7	Moderate (B)	Global	95	10	12	308	12
8	Severe (B)	No injury	99	0	0	213	0
9	Severe (A)	Punctate	138	5	2	258	5
10	Severe (A)	Watershed	107	6	7	298	6
11	Severe (B)	Central	91	5	0	241	10
12	Severe (B)	Central	95	6	6	191	8
13	Severe (A)	Global	75 (HT)	11	9	283	11
14	Severe (B)	Global	157	12	6	336	11
15	Severe (B)	Global	71 (HT)	10	10	377	12

HIE: Hypoxic-ischemic injury; Moderate HIE (A): without seizures (B): with seizures; Severe HIE (A): no signs of brainstem dysfunction (B): signs of brainstem dysfunction; DWI: Diffusion-weighted imaging; MR1: first/early magnetic resonance imaging; MR2: second/late magnetic resonance imaging; HT: Hypothermia.

The localization and extension of hypoxic-ischemic injury in early and late scans were highly correlated. Hypoxic-ischemic injury scores from conventional sequences (T1/T2) in the early MRI correlated with the scores of the late MRI (Spearman ρ = 0.940; p < .001) as did the scores between DWI in early scans and conventional images in late MR studies (Spearman ρ = 0.866; p < .001).

## Discussion

The high correlation between the two sequential MR studies suggests that in infants with moderate or severe HIE treated with hypothermia, conventional and diffusion MR images performed around the fourth day of life can accurately depict hypoxic-ischemic lesions seen in later scans performed during the second week of life. This is relevant because in these first days of life MRI may provide important information for clinical prognostication and parental discussion.

We know from the randomized control trials in hypothermia that almost 2/3 of deaths followed end of life decisions. In a recent study the mean age ± SD of death in infants with HIE following end of life decisions was 64 ± 51 hours [18]. In clinical practice there is a window of opportunity for decision making, as described by Wilkinson [19]. When the clinical status of a baby is severe and there is enough certainty about the prognosis, it is considered ethical and appropriate for parents and physicians to take advantage of the window to withdraw life-sustaining treatment. Prognosis in HIE is based on clinical, neurophysiological and neuroimaging findings. However, several studies have shown that therapeutic hypothermia changes the prognostic value of clinical grading of neonatal encephalopathy [20] and aEEG monitoring [21,22]. Gunn et al. suggested that infants with moderate encephalopathy on day 4 might have a more favourable prognosis after hypothermia treatment than expected after standard care [20]. In a recent study, Thoresen et al. showed that hypothermia changes the predictive value of an early abnormal background and that hypothermia-treated infants can still develop normally as long as the aEEG recovers before 48 hours of life [21,22].

Qualitative evaluation on conventional or diffusion MR images during the first 48 hours may underestimate hypoxic-ischemic lesions in the presence of significant brain injury [23]. Due to these limitations other quantitative techniques such as proton magnetic resonance spectroscopy (1H-MRS) and quantitative diffusion parameters have been developed [24-26]. However in clinical practice their use in HIE infants during the first 48 hours of life is limited: very early imaging of sick newborns with HIE is technically challenging, and their role in perinatal HIE treated with hypothermia is still being evaluated. On the other hand, because hypothermia is a standard of care in developed countries, most of the brain MR studies are usually performed after the third day of life when rewarming of the infants has been completed. Although it is still unclear how cooling to 33–34°C for 72 h impacts on the evolution of early MR brain images, a recent RCT suggests that hypothermia does not seem to delay the appearance of brain abnormalities [27]. Moreover, an excellent correlation between brain injury in MRI and outcome of death and disability at 18 months of age has been demonstrated in these trials [27-29].

In clinical practice if there are doubts about prognosis or redirection of care is considered, MR performed in the first days of life may be very valuable in infants with significant HIE. Although it is not essential and not always available, it is especially useful if there are inconsistencies with the other prognostic tools as neurological examination or neurophysiological studies. Therefore, it is essential to determine the optimal earliest timing of imaging in asphyxiated newborns treated with cooling to most accurately define the degree of brain injury sustained and predict neurological outcome. According to the high correlation between early and late sequential MRI we have observed, scans performed around the fourth day of life can be useful for clinicians and parents for prognostic proposes and for redirection of care.

**Figure 1:**
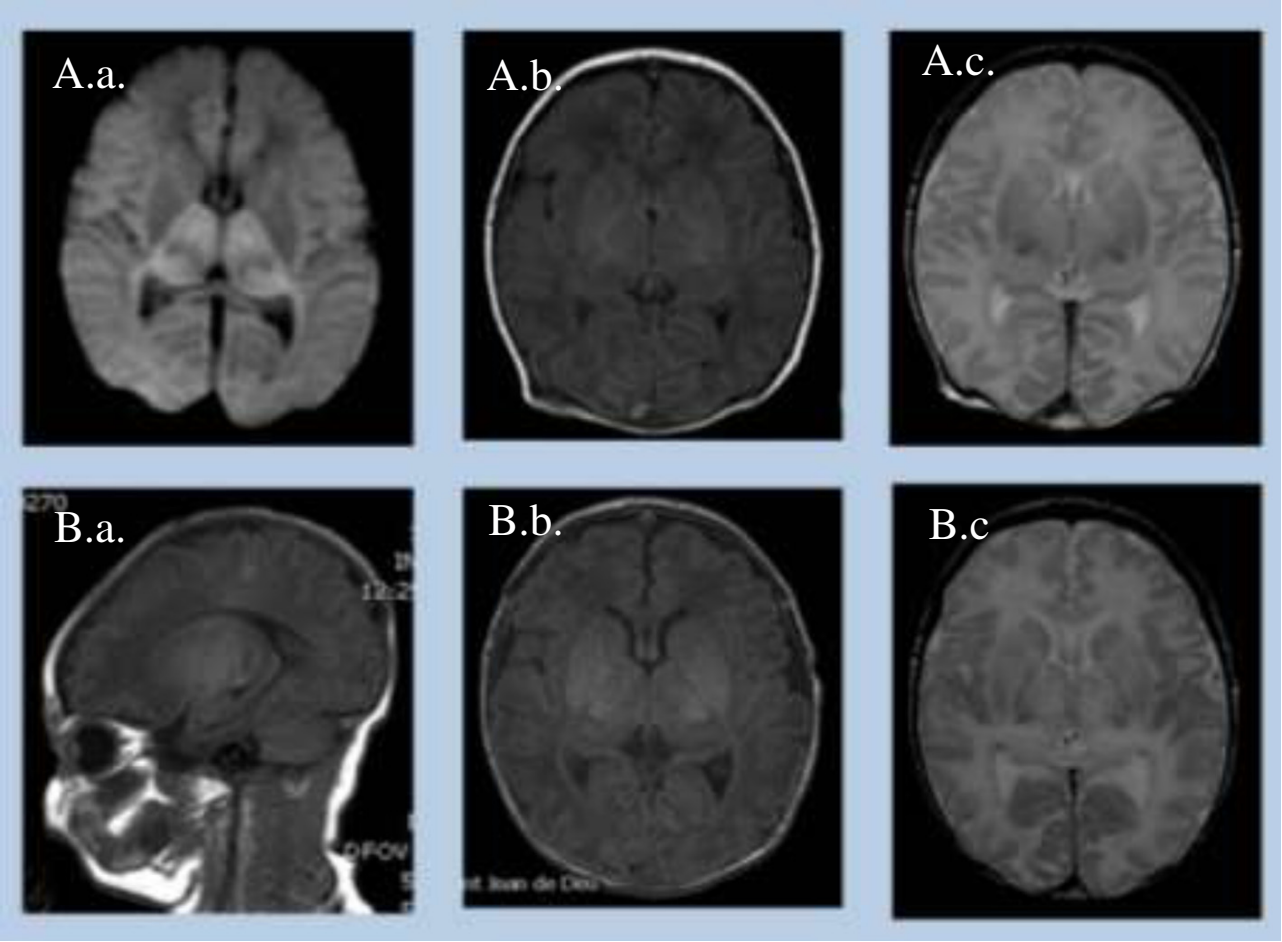
**Sequential MR studies in a newborn with severe HIE and a central pattern of injury (ID number 12). A**. Early MR study. **A.a.** Axial diffusion-weighted image: restricted diffusion in the thalami; **A.b.** Axial T1 weighted images: abnormal high intensity within the basal ganglia and thalami (BGT) and loss of the normal intensity of the posterior limb of the internal capsule. **A.c**. Axial T2 weighted image. Low and high signal intensity within the basal ganglia and thalami. **B**. Late MRI study confirmed the previous findings. Sagittal **(B.a.)** and axial **(B.b.)** T1 weighted images showing an abnormal widespread high intensity of BGT and confirming the absence of the normal intensity of the posterior limb of the internal capsule. Cortical highlighting around central fissure. **B.c.** Axial T2 weighted images with heterogeneity of the signal in BGT more evident than in the early MRI.

A novel and interesting aspect of our study is the two sequential MR scans performed in 15 newborns for hypoxic-ischemic brain injury assessment including DWI in the early examinations. In our study conventional sequences were independently evaluated from DWI. All MR scans have been performed with the same equipment and imaging protocol with no variability in the sequences. Images were analysed by two paediatric neuroradiologists who were blinded to any clinical data or the order of the study. It is important to highlight that we didn’t find any false negative in the patients with serial MR studies. Findings between the first and second conventional MR scans were similar in 9 cases. In the 6 newborns left in which differences were noted in scores between the two sequential scans, there were no serious abnormalities of the first scans that normalized in the second week. In 3 of these patients differences in scores were because PLIC was scored as doubtful in the first scan and absent in the late scan (patient number 7, 12 and 15). Two patients with central pattern (patients 6 and 13) scored higher because mild abnormal signal intensity in the cortex was seen in the late scan that was missed in the early scan. One patient (patient 11) was classified as having a moderate central pattern in the conventional sequences of the first scan at 74 h (score of 5) and as having severe central pattern in the late scan (score of 10). Except for this infant, no serious abnormalities in the early scans that would have change the prognosis were missed. In one study comparing sequential MR scans, Wintermark *et al.* suggest that MRI scans obtained in the second and third days of life during hypothermia may predict later brain injuries in asphyxiated newborns. They could only obtain two sequential MR scans in 9 newborns and the number of patients with brain injury in that study was small (N = 4) [13]. Our findings in the 15 newborns with two sequential MR scans are coincident with the results reported by Wintermark *et al*. (Figure 1).

There are several limitations to our study that need to be addressed. First of all, the small number of newborns with both MR studies, although there were no differences in neonatal characteristics between newborns without MRI study. The ratio between infants with severe and moderate HIE may represent a selection bias. Two thirds of our patients were referred form other centers and this study was conducted during the period when implementation of cooling for treatment of HIE infants was started at our country. Most of the infants with severe HIE were outborn (Table 1). In severe cases is easier to depict lesions in early scans and this could explain the good correlation between the early and the late MR scans. However the most severely ill infants are the ones that died and obviously they don’t have both MR studies and are not included in our analysis. We couldn’t evaluate the correlation with spectroscopy findings as it was not available in all the neonates. Finally the main variable used as gold standard to confirm brain injury in these newborns was late MRI evidence of brain hypoxic–ischaemic injury instead of neurodevelopmental outcome or autopsy data.

## Conclusions

We found a high correlation between early and late sequential MRI in HIE infants treated with hypothermia. Our data suggest that MRI in the first days of life may be a useful prognostic tool for clinicians and parents and can help in medical issues such as end of life decisions. Further studies evaluating the outcome of these patients and the use of advance imaging techniques, may help to determine the timing for imaging in this population.

## Abbreviations

MRI: Magnetic resonance imaging; HIE: Hypoxic-ischemic encephalopathy; TH: Therapeutic hypothermia; aEEG: Amplitude-integrated electroencephalography; DWI: Diffusion weighted images; BGT: Basal ganglia and thalamus; PLIC: Posterior limb of the internal capsule; WM: White matter; H-MRS: Magnetic resonance spectroscopy.

## Competing interest

The authors declare no conflicts of interest. TA received a grant from the Spanish Neonatal Society for the development of this work.

## Authors’ contributions

TA, reviewed data collection, prepared and wrote the first draft of this paper, and coordinated manuscript revisions and submission. MA, reviewed data collection, revised and edited the manuscript. MR and JM scored MR images and revised and edited the manuscript data collection. GA, reviewed data collection and edited the manuscript. AGA planned and designed the study; revised and edited the manuscript. All the authors have seen and approved the final version.

## Pre-publication history

The pre-publication history for this paper can be accessed here:

http://www.biomedcentral.com/1471-2431/14/177/prepub
